# 

**Published:** 1895-07-06

**Authors:** 


CONTENTS.

Snake Poisons and Antidotes - 229

Dr. Bentoul's Rhapsody - 230

The Classification of Aneurisms. By A. Pearce Gould, F.R.C.S. - 231

The Alleged Increase of Heart Disease - 232

Medical Progress and Hospital Clinics—

Nævi - 235

Progress in Medicine.—Diseases of the Lungs—Gout - 236

Progress in Surgery.—Cerebral Surgery - 239

Therapeutic Notes.—Benzine Poisoning—Calomel and Potassium Bromide - 240

New Appliances and Things Medical - 240

Annotations.—The Notification of Measles—Huxley's Aim in Life—Medical Defence and Protection - 233

Notes and News - 234

The Institutional Workshop—

Brixham Cottage Hospital - 241

Dundee Royal Infirmary—New Operation Theatre - 241

The Story of the Insane from Year to Year - 242

Hospital Meetings - 243

Metropolitan Hospital Sunday Fund - 244

Practical Departments - 244

Scraps and Gleanings - 244

The Book World of Medicine and Science - 245

EXTRA SUPPLEMENT.—THE NURSING MIRROR.

News from the Nursing World.—The Royal Visit to St. Bartholomew's—At the Royal Free Hospital—Trials of Training—Nurse or Schoolmistress?—Inexcusable Silence—Unpractical Suggestions—Preparation for Casualties—A Good Report—A Model Emergency Hospital—A Nurses' Guild—Short Items - xci

Nursing in America.—The Convention of Superintendents and Members of the Association - civ

Elementary Anatomy and Surgery for Nurses. By W. McAdam Eccles, M.D., M.S., F.R.C.S. XXIV.—The Nervous System - xciii

Notes and Queries - xcii

Where to Go—Appointments - xciv

Holidays and Health - xcv

Queen Victoria Jubilee Institute - xcvi

The Book World for Women and Nurses - xcvi

